# The Effect of Ethanol on Telomere Dynamics and Regulation in Human Cells

**DOI:** 10.3390/cells7100169

**Published:** 2018-10-15

**Authors:** Tomer Harpaz, Heba Abumock, Einat Beery, Yonatan Edel, Meir Lahav, Uri Rozovski, Orit Uziel

**Affiliations:** 1 The Felsenstein Medical Research Center, Rabin Medical Center, Petah-Tikva 49100, Israel; tharpaz@greenpeace.org (T.H.); shhebaab@clalit.org.il (H.A.); einatb@clalit.org.il (E.B.); mlahav@post.tau.ac.il (M.L.); Rozovski.uri@gmail.com (U.R.); 2 Sackler School of Medicine, Tel-Aviv University, Tel Aviv 69978, Israel; yonatanad@clalit.org.il; 3 Medicine C, Rabin Medical Center, Petah-Tikva 49100, Israel; 4 Institute of Hematology, Davidoff Cancer Center, Rabin Medical Center, Petah-Tikva 49100, Israel

**Keywords:** telomeres, ethanol, shelterin, telomerase, acetaldehyde

## Abstract

Telomeres (TLs) protect chromosome ends from chromosomal fusion and degradation, thus conferring genomic stability, and play crucial roles in cellular aging and disease. Recent studies have found a correlation between environmental, physiological and even mental stresses on TL dynamics in humans. However, the causal relationship between stress and TL length and the molecular mechanisms underlying that relationship are far from being understood. This study describes the effect of moderate concentrations of ethanol, equivalent to social drinking, on human TL dynamics and partially elucidates the mechanism mediating this effect. The exposure of Immortalized human foreskin fibroblast, primary human foreskin fibroblast and human hepatocellular carcinoma cells to 25 mM ethanol for one week moderately shortened telomeres in all cells. Similar TL shortening was obtained following cells’ exposure to 25 µM acetaldehyde (AcH) and to a much lower extent after exposure to 4-methylpyrazolean, an inhibitor of alcoholdehydrogenase, suggesting that AcH plays a key role in ethanol-dependent telomere shortening. Telomerase activity was not involved in this effect. TRF2 and several TRF2 binding proteins increased their binding to TLs after ethanol treatment, implying their involvement in this effect. The methylation status of several sub-telomeric regions increased in response to EtOH exposure. Gene expression profiling showed distinct patterns in cells treated with EtOH and in cells recovered from EtOH. In addition to cellular ageing, the described telomere shortening may contribute to the carcinogenic potential of acute alcohol consumption; both are associated with the shortening of TLs and provide new insights regarding the moderate consumption of alcohol referred to as “social drinking.”

## 1. Introduction

Telomeres (TLs), (TTAGGG)n elements found at the ends of each linear chromosome, distinguish chromosome ends from being recognized as double-strand breaks (DSBs), thus preventing a false response of DNA damage repair (DDR) that would otherwise result in the joining of two chromosomal ends [1,2]. Telomeres erode in each DNA replication until reaching a threshold that signals the cell to enter senescence. This shortening is attenuated by telomerase, a reverse transcriptase ribonucleoprotein that elongates TLs [3,4]. TLs are accompanied by the shelterin complex comprised of six core proteins: TRF1, TRF2, RAP1, POT1, TIN2 and TPP1. The shelterin complex retains TLs stability, inhibits DDR pathways and regulates TLs length by providing positive/negative access to telomerase [5]. Additionally, a plethora of other proteins, mostly involved in DNA damage repair processes, are connected indirectly to telomeres. These proteins include: tankyrase 1 and 2, poly (ADP ribose) polymerase (PARP), meiotic recombination 11 homologue (MRE11), the RecQ-like helicases WRN (Werner’s syndrome protein) and BLM (Bloom’s syndrome protein), Ku70, Ku86, DNA-dependent protein kinase (DNA-PK), ataxia-telangiectasia mutated (ATM), Rad3-related (ATR), excision repair cross complementing 1 (ERCC1), RNA-polymerase σ 70 factor (XPF) and RAD50 [6].

Ethanol (EtOH) is a type of a psychoactive drug having intoxicating and recreational effects [7,8,9,10,11,12]. Three enzymatic pathways oxidize ethanol into acetaldehyde (AcH): alcohol dehydrogenase (ADH), responsible for ~90% of EtOH oxidation in the body [13,14]; Cytochrome P450 2E1 (CYP2E1) and catalase. AcH is a highly unstable compound that quickly forms free radicals [15,16]. It is suggested that AcH intoxication is responsible for the unpleasant “hangover” [17,18,19] symptoms after alcohol consumption.

Over the past few decades, accumulating studies have shown that long-term moderate consumption of alcohol has several beneficial effects, mainly the reduction of the risk of illnesses including cardiovascular diseases [20,21,22], diabetes and osteoporosis [23,24], Alzheimer’s [25,26] and cancers [27,28,29,30].

“Moderate consumption” varies between different countries and individuals according to age, gender, weight and body stature, genetics and other factors [15,31]. “Moderate drinking” is considered 1–3 drinks per day, which are 4–30 mM [32].

Several studies focused on the effects of mental stress on telomere length in humans [33]. However, no study has examined the effects of ethanol on human telomeres. In a previous study looking for the effects of various stresses on yeast [34], EtOH exposure markedly increased telomere length. That effect was mediated by the Rap1-related pathway, which in yeast binds directly to telomeres and also to the ATM/ATR-related pathway. Following these results, we sought to characterize the possible effect of EtOH, equivalent to social drinking, on human telomere dynamics and elucidate the mechanism mediating this effect.

## 2. Methods

### 2.1. Experimental System and Growth Conditions

The study employed three cell types: human foreskin fibroblasts (hFF-IM), immortalized by an ectopic expression of hTERT (human telomerase reverse transcriptase); primary human foreskin fibroblasts (hFF-P) (both provided kindly by Prof. Skorecki’s lab, the Technion, Haifa) and human hepatocellular liver carcinoma cell line (HepG2) (kindly provided by Dr. Zemel, FMRC, Rabin Medical Center and Tel Aviv University). All cells were grown with growth media based on DMDM supplemented with 20% FBS, 1% Penicillin/Streptomycin and 1% Glutamine. Cells were incubated in the presence of 5% CO_2_, with 95% humidity at 37 °C.

### 2.2. Cell Viability Assay

Cell viability was determined by the colorimetric assay WST-1, whereby 5 × 10^3^ cells/well cells were cultured in a 96-well plate with 200 µL of growth medium. After six days of growth, 20 µL of WST-1 reagent was added to each well and after 1 h absorbance was measured by an ELISA reader at 480 nm, with a reference wavelength of 600 nm. Clear medium was used as a background.

### 2.3. Southern Blot and Mean TRF Measurement

Genomic DNA was extracted (ArchivePure; 5-prime, Gaithersburg, MD, USA) according to the manufacturer’s instructions and quantified (NanoDrop; Thermo Scientific, Waltham, MA, USA). DNA concentrations were mostly ~1 µg/µL and the A260/280 ratio was about 1.8. The A260/230 ranged between 2.0 and 2.2. Five micrograms of DNA were loaded on each lane of a 0.8% agarose gel prior to the Southern blot procedure.

Southern blot was performed with the TeloTAGGG™ Telomere Length Assay kit (Sigma-Aldrich, St. Louis, MO, USA) according to the provided manual. To calculate Terminal Restriction Fragments (TRF), signals were scanned and quantified by the Quantity One software (Versadoc MP; BioRad, Hercules, CA, USA) and calculated according to the following equation:

(1) $$\frac{\sum \left({\mathrm{OD}}_{i}\right)}{\sum ({\mathrm{OD}}_{i}/L_{i})},$$

where OD_i_ is the chemiluminiscent signal and L_i_ is the length of the TRF at position i.

### 2.4. Telomeres Repeat Amplification Protocol (TRAP)

Telomerase activity (TA) was measured by the TRAP assay [35] (TRAPeze; Millipore, Burlington, MA, USA). 10^6^ cells were harvested and lysed using the supplied lysis buffer. Total protein concentration was determined by a Bradford assay. Protein samples of 100 ng were incubated for 30 min at 30 °C and subsequently a standard PCR protocol was used. Samples were then separated by PAGE, the gel was stained (GelStar; Cambrex, East Rutherford, NJ, USA) and TA was visualized and quantified with Quantity One software (Versadoc; MP; BioRad). The activity of telomerase was also calculated by using a real-time PCR-based assay [36] (QTD Kit; Allied Biotech, Taipei, Taiwan). The PCR program included 20 min at 25 °C, 10 min at 95 °C and 40 cycles of 30 s at 95 °C, 30 s at 60 °C and 30 s at 72 °C. TA was calculated using TSR standard curve equation and C_t_ values of the samples.

### 2.5. DNA Methylation

The DNA methylation status of the sub-telomeric region was determined by Nucleix©. The assay is based on the amplification of that region in specific chromosomes followed by pyrosequencing. A consensus sequence of subtelomeric regions was based on common sequences in the sub-telomeric regions according to [37].

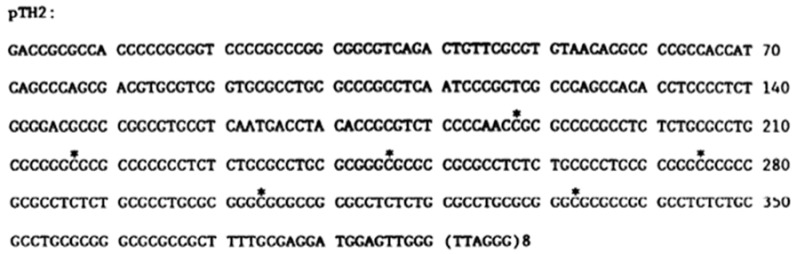



The asterisks mark the repetition of a specific sequence characterizing the subtelomeric regions.

### 2.6. Gene Expression Profiling

The gene expression profiling was performed at the Bioinformatic Unit, Tel Aviv University. hFF-IM cells were harvested, RNA was immediately extracted according to the manufacturer’s protocol (RNeasy mini; Qiagen, Hilden, Germany) and the concentration was determined (Nanodrop; Thermo Scientific), visualized on an agarose gel to follow its stability. The RNA was transcribed and hybridized with 20,000 genes (Affymatrix, Santa Clara, CA, USA). The analysis of the results was made by the Bioinformatics unit.

### 2.7. Western Blot

Cells were lysed using a protein lysis buffer and protein contents was measured by the Bradford assay, as described above. One hundred micrograms of each sample were loaded onto a gel for PAGE. The gel was then transferred to a nitrocellulose membrane that was subsequently hybridized with 2 µg of anti-γH2A.X antibody (Cell Signaling, Danvers, MA, USA) and anti-GAPDH (Santa Cruz Biotechnology, Dallas, TX, USA) as a reference gene. Then, membranes were hybridized with a fluorescent-labeled secondary antibody (LI-COR, Lincoln, NE, USA) and protein levels were visualized and quantified (Odyssey IR imaging system, LI-COR).

### 2.8. Chromatin Immunoprecipitation (ChIP) Dot Blot

The levels of the shelterin complex members binding to TLs were followed by the ChIP assay (EZ-ChIP; Millipore) according to the provided manual. Briefly, the DNA was cross-linked to its bounded proteins using formaldehyde. The cells were then collected and sonicated to shear chromatin to a size of 200–1000 bp (validated by following the separation of the DNA in an agarose gel). The immunuselection was made using antibodies for each of the six Shelterin proteins: 1 µg of anti-TRF1 (Santa Cruz Biotechnology), 1 µg of anti-TRF2 (Santa Cruz Biotechnology), 1 µg of anti-RAP1 (Santa Cruz Biotechnology), 1 µg of anti-POT1 (Santa Cruz Biotechnology), 2 µg of anti-TPP1 (Abcam, Cambridge, UK) and 4 µg of anti-TIN2 (Protein Tech, Rosemont, IL, USA). Subsequently, the DNA‒protein‒antibody complexes were precipitated by binding to protein-A-agarose beads. The DNA was then reverse-cross-linked during incubation at 65 °C. RNA and protein were degraded by RNAse and proteinase K, respectively, and the DNA was purified (QIAquick PCR Purification, Qiagen). Eluted samples were dot-blotted on positively charged Nylon membrane (Roche, Basel, Switzerland). Subsequent steps were similar to that of the Southern blot method.

### 2.9. Cell Exposure to Various Treatments

All cells were split two times a week. After every split, they were provided with a medium and exposed to the following treatments:

1. 25 mM EtOH for two days.
2. 25 mM EtOH for one week.
3. 25 µM AcH for two days or one week.
4. 25 mM EtOH + 2 mM 4-MP.
5. Recovery: Comprised of a first week of standard EtOH treatment followed by a second week of EtOH-free medium.

## 3. Results

### 3.1. The Addition of 125 mM EtoH Does Not Affect Cell Viability

**Figure 1:**
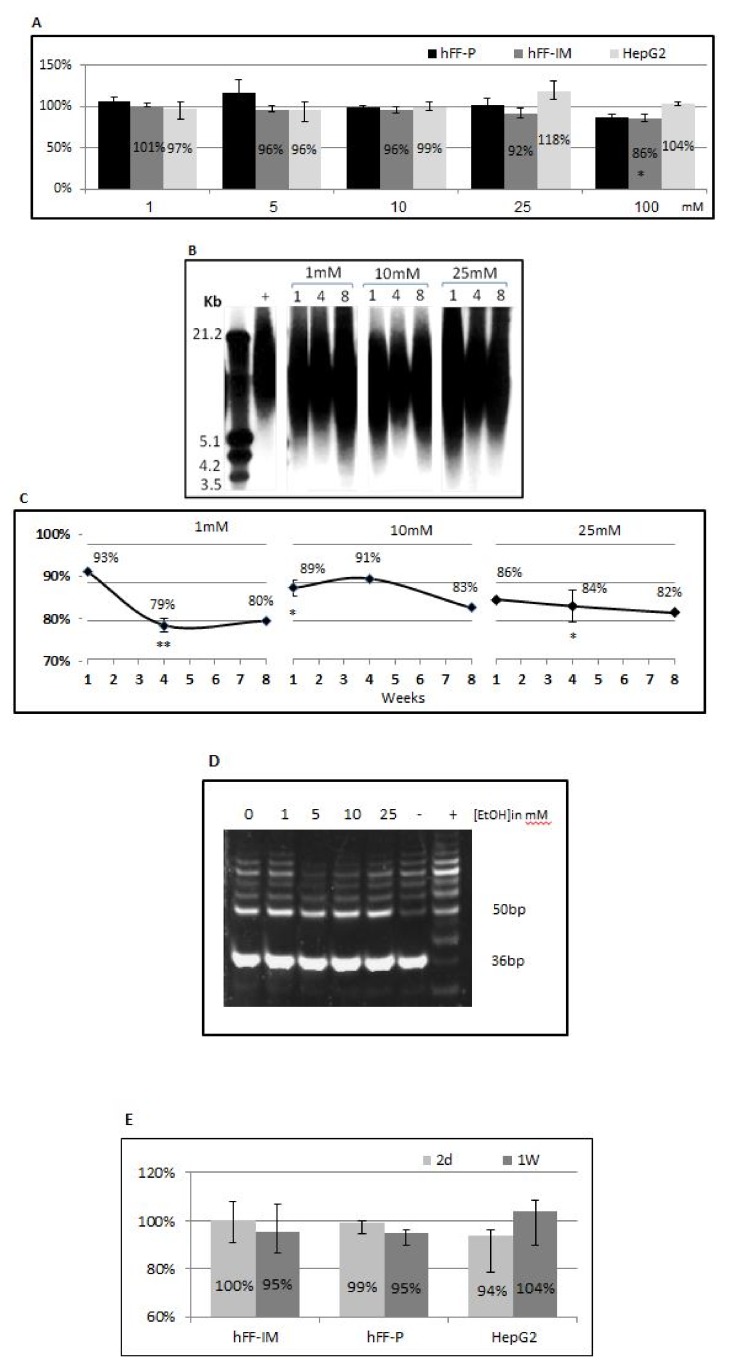
Cell viability and telomere length of various cell lines after treatment with EtOH. (**A**) Three cell lines, hFF-p, hFF-IM and HEPG2, were exposed to 1–100 mM EtOH for six days and their proliferation was measured by Wst-1 assay. Percentages represent values relative to the untreated control of each cell line. hFF cells were treated with 1, 10 and 25 mM EtOH for one, four and eight weeks and their telomeres were measured by Southern blot. (**B**) A representative example of Southern blot with DNA isolated from pHFF cell samples. +depicts TL of control untreated cells. (**C**) Quantitation of the Southern blot results showing the mean TRF values of treated vs. untreated. * pV < 0.05; ** pV< 0.01. (**D**) TA of hFF-IM cells in response to EtOH exposure: hFF-IM cells were treated with 1–25 mM EtOH for two days or one week and their TA was measured by the TRAP assay, A representative TRAP assay example. (**E**) TA of all three cell lines in response to EtOH exposure. pV < 0.01.

The cell viability in response to EtOH was tested using the WST-1 assay (Figure 1A). EtOH in concentrations of 1–25 mM did not significantly affect the proliferation of the following cells: hFF-IM, primary foreskin fibroblasts (hFF-P), serving as control for hFF-IM, and A human hepatocellular liver carcinoma cell line (HepG2) known to possess liver cell morphology. However, exposure to 100 mM EtOH resulted in a 15% decrease in proliferation (pV < 0.05). These data may suggest that cell viability is not likely to be afflicted at the alcohol concentration corresponding to moderate drinking, while higher EtOH levels may be toxic to cells. Therefore, we used low EtOH concentrations (1–25 mM EtOH) for the rest of the study and a concentration of 25 mM EtOH for a single week was chosen as the optimal one. To explore the putative mechanisms that mediate the effects of EtOH on TL length, a time point of two days (in which TL length was not yet affected) was used.

### 3.2. EtOH Shortens Telomeres

hFF-IM cells were exposed to EtOH for eight weeks within the “moderate drinking” concentrations. Cells were sampled along several time points and the length of TLs were measured (Figure 1B,C). The results indicate that EtOH induces TL shortening, which can be seen from the first week of exposure and was significant after four weeks. That exposure period led to a significant decrease in TL length of 22% and 16% with 1 mM and 25 mM treatments, respectively (pV < 0.01). Of note, telomeres were not shortened in the cells due to the presence of ectopically expressed telomerase (not shown).

### 3.3. EtOH Effect on TL Length Is Not Mediated by Telomerase Activity

Telomerase activity (TA) was measured in all three cell lines (Figure 1D,E). The results showed no difference in TA between treatment with 1–25 mM EtOH and control cells, suggesting that TA is not involved in TL shortening by EtOH.

### 3.4. TRF2 and RAP1 Binding to Telomeric Regions Are Affected by EtOH

**Figure 2:**
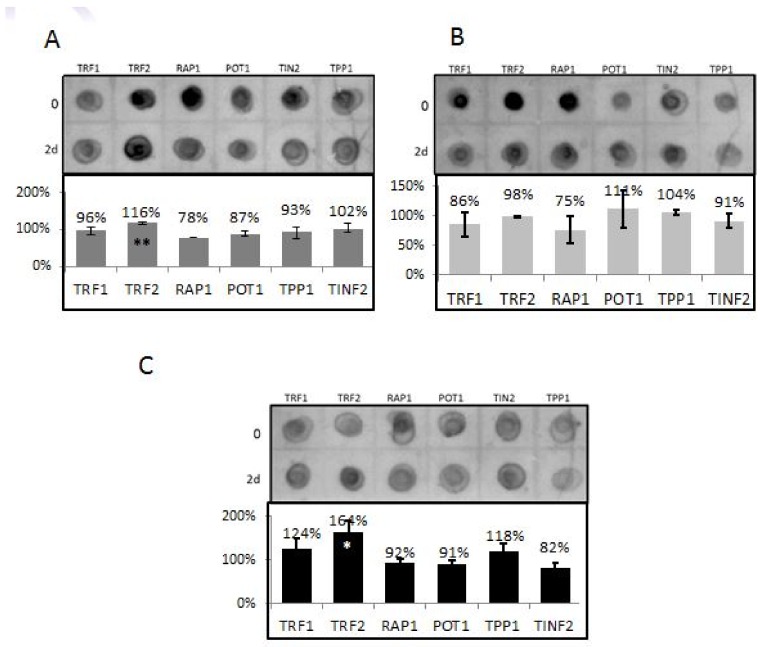

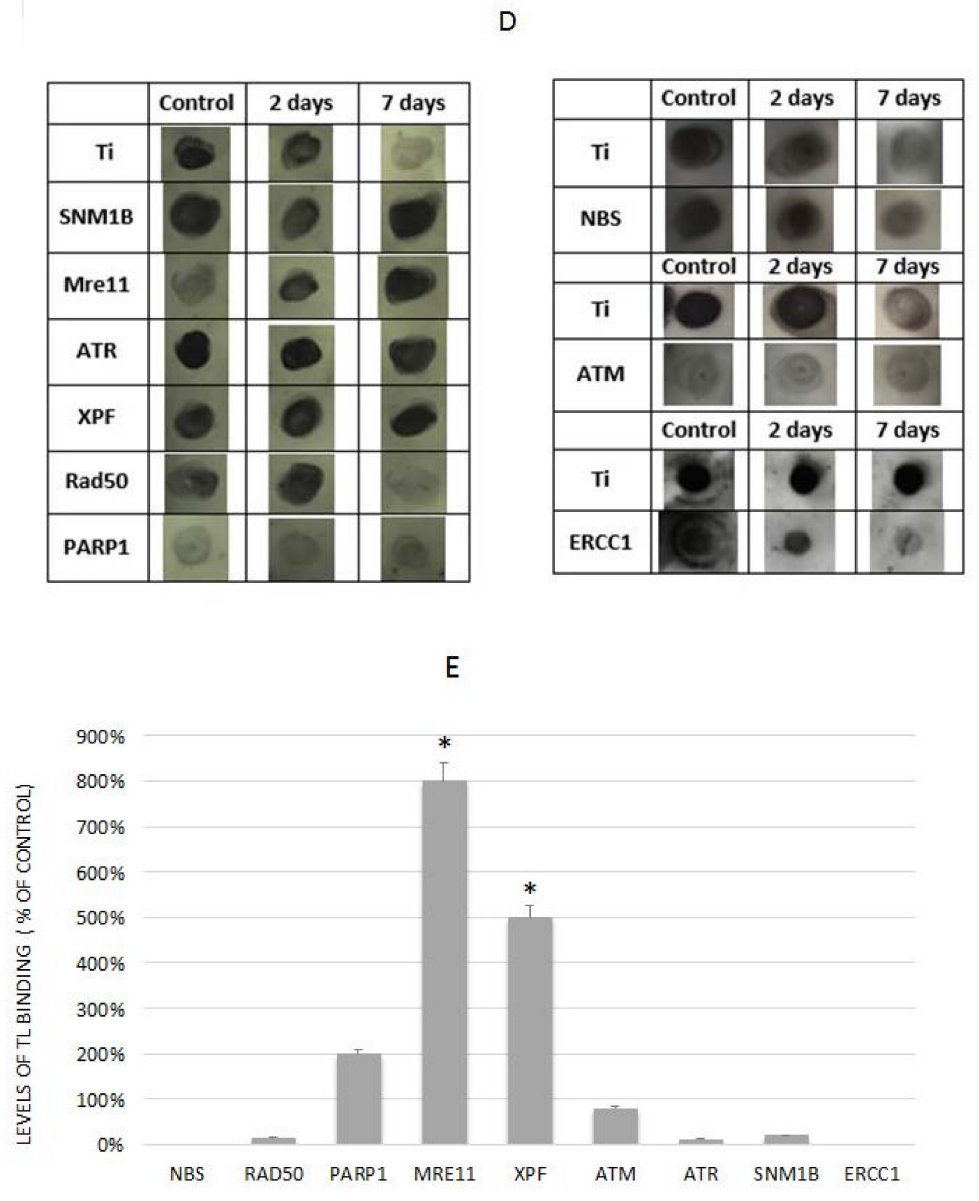
ChIP dot blot of all six Shelterin proteins, TRF1, TRF2, RAP1, POT1, TIN2 and TPP1, in three different cell lines. The upper part of each panel shows a representative dot blot and the lower represents three independent experiments. All cells were exposed to 25 mM EtOH for two days and subjected to the ChIP dot blot assay. Values indicate the signal intensity relative to the total input samples signals. (**A**) Primary hFF; (**B**) hFF-IM; (**C**) HepG2 cell line. * pV < 0.05 ** pV < 0.01. (**D**) The level of binding and the total cellular levels of TRF2 binding proteins to telomeres in hFF-IM cells assessed by ChIP dot blot. Examples of the various dot blots. (**E**) Quantitation of signal intensities.

A second player in TL length regulation is the shelterin complex. To study its potential involvement in the shortening of TL obtained by EtOH exposure, the binding of all six proteins of the shelterin complex to TLs was evaluated. Cells were exposed to 25 mM EtOH for two days and subjected to the ChIP dot blot assay (Figure 2). Analysis of the results demonstrated a significant increase in TRF2 binding to TLs both in hFF-P and in HEPG2 cell lines after EtOH exposure. In contrast, RAP1 binding was decreased in all cell lines. There was no significant change in the binding of all other shelterin members to TLs in response to EtOH treatment.

### 3.5. Differential Binding of TRF2-Associated Proteins to Telomeres

**Figure 3:**
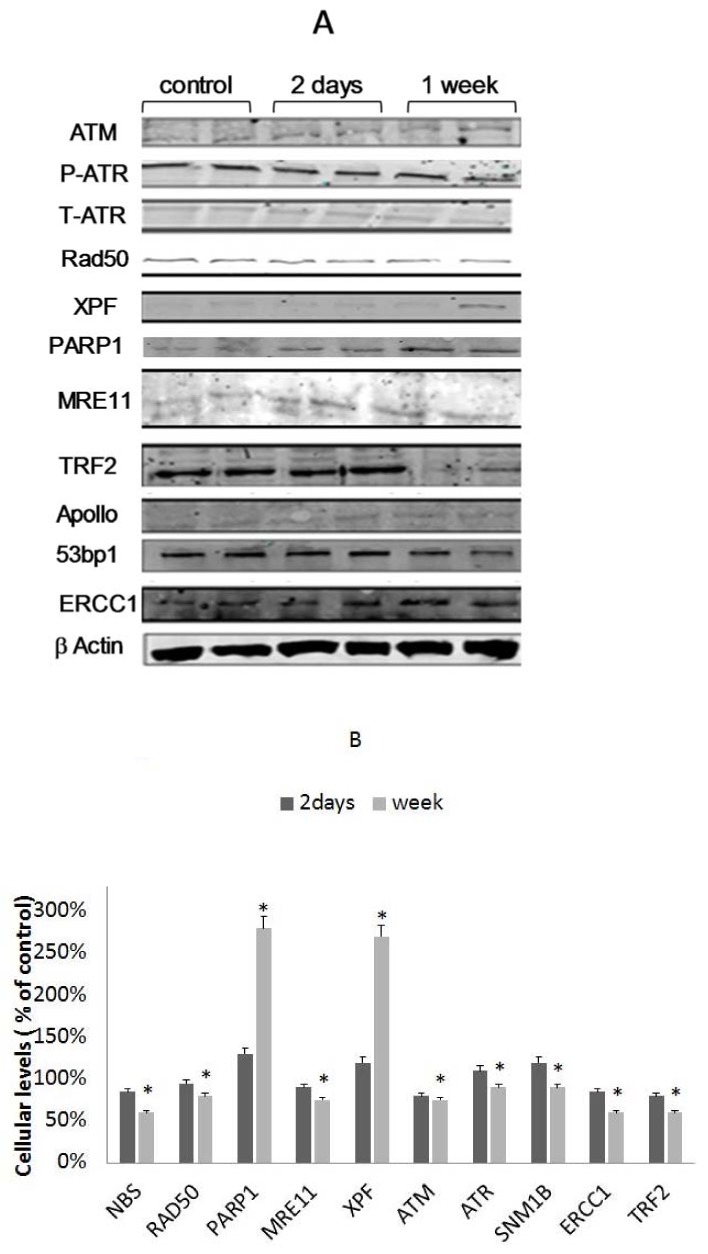
The cellular levels of TRF2 binding proteins. pHFF cells were exposed to 25 mM ethanol for two days and the level of the TRF2 binding proteins was assessed by Western immunoblot. (**A**) A representative example of the Western blots; (**B**) quantitation of A, performed by the VersaDoc densitometer quantitation apparatus. * pV < 0.05.

TRF2 is known as a major regulator of telomere length. In order to further elucidate the mechanism by which EtOH-dependent telomere shortening occurs, we analyzed the levels of binding of various TRF2-associated proteins in response to EtOH treatment prior to the induction of telomere shortening. We measured the cellular levels and the level of binding of each protein to telomeres after two days of 25 mM EtOH treatment. The results are shown in Figure 3A,B. All of the examined proteins have increased their binding to telomeres after two or seven days of exposure, while XPF and PARP1 were maximally elevated. These results also substantiate the biological significance of the increase in TRF2 binding to telomeres after EtOH treatment.

### 3.6. The Involvement of XPF in EtOH-Dependent Telomere Shortening

**Figure 4:**
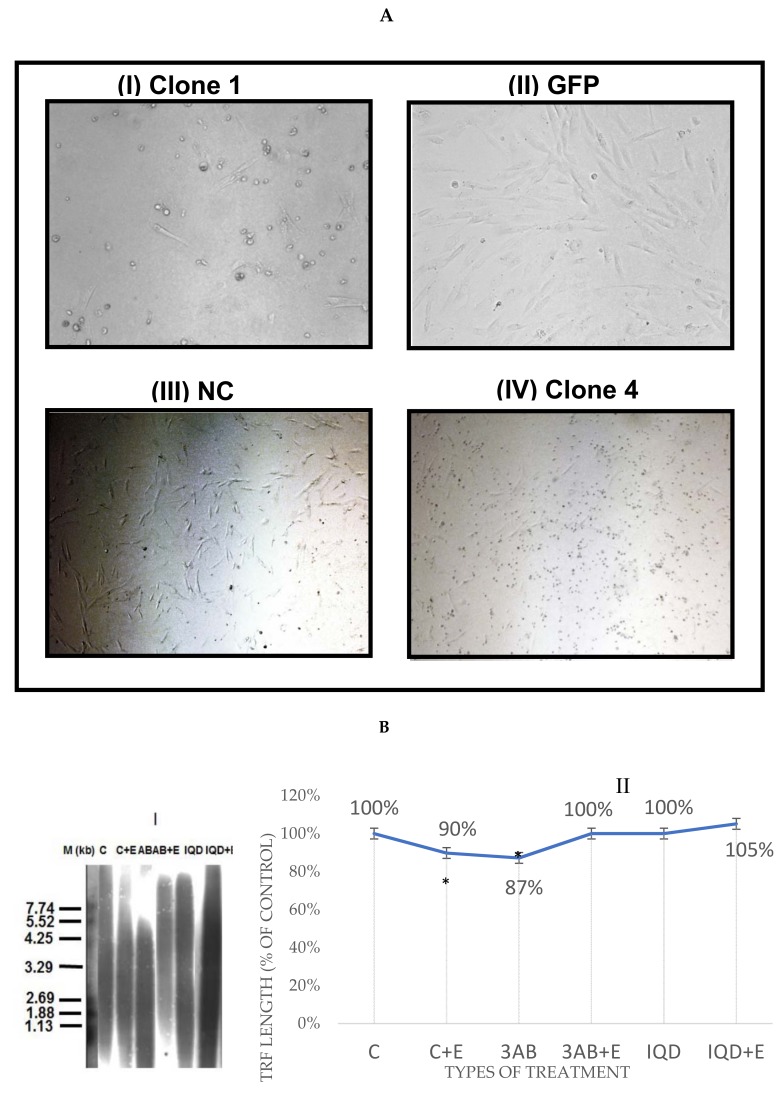

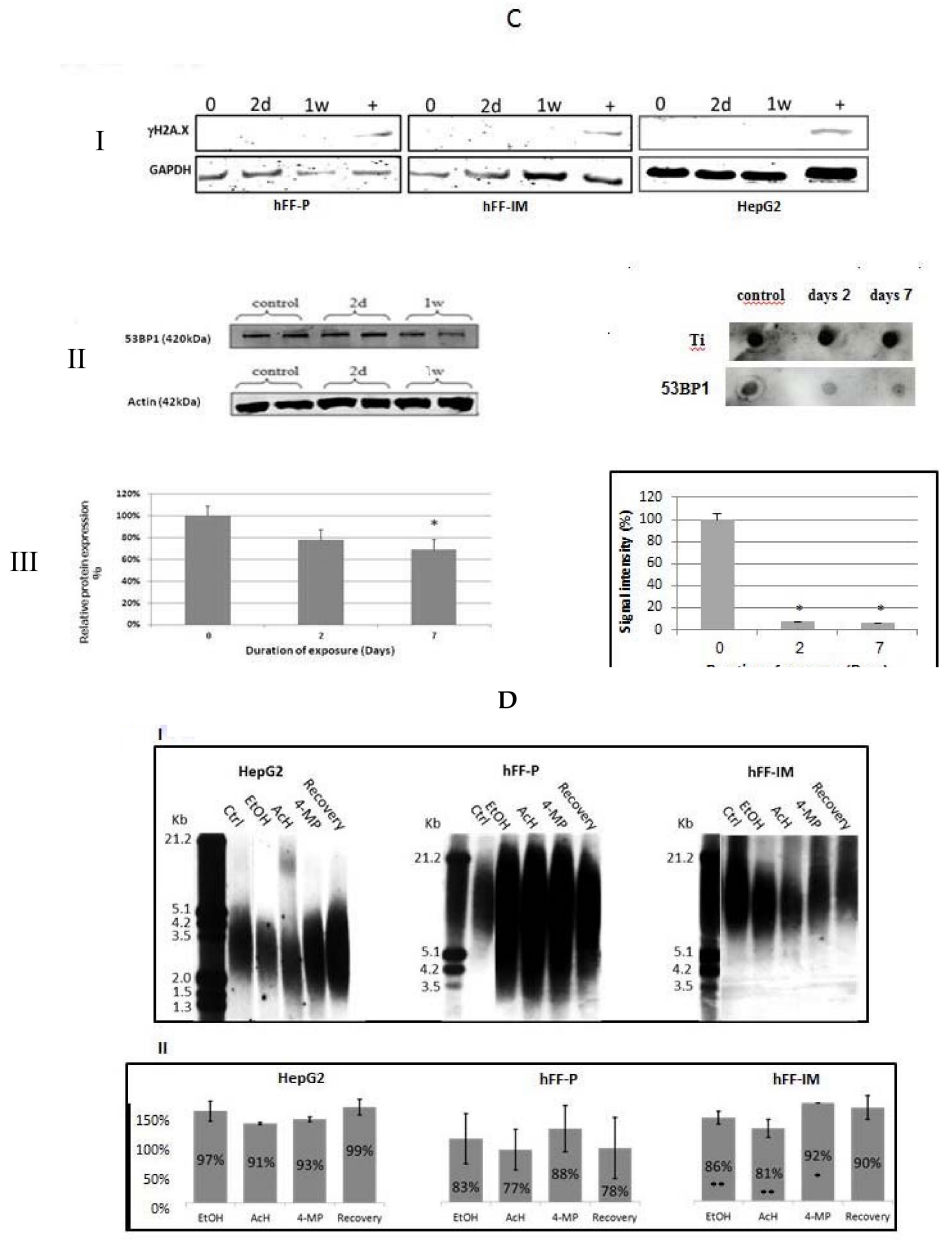
Viability of Primary hFF cells after knockdown of XPF. (**A**) XPF expression was knocked down by stable transfection with a specific shRNA plasmid. (I) an example of one transfected clone; (II) a control sample transfected clone with shRNA against GFP; (III) a control sample transfected clone with a scrambled shRNA (IV) a control intact sample. (**B**) (I) Telomere length in Primary hFF cells treated with PARP1 inhibitors as measured by Southern blotting. Cells were treated with EtOH (E) and both PARP1 inhibitors for seven days (3AB–2 mM 3-Aminobenzamide, IQD- 0.1 mM 1,5-Isoquinolinediol). C: Control cells. C + E: cells treated with 25 mM ethanol as above. IQD + E: cells treated with 1,5-Isoquinolinediol and 25 mM ethanol. Each lane contains a smear that represents the varied length of the TLs in the specific sample. (II) Quantitation of Southern blot experiments showing the mean TRF values of treated vs. untreated cells calculated as percentage of control. * *p* < 0.001. (**C**) γH2AX and 53bp1 expression in all cells after treatment with 25 mM EtOH for two days and one week. Cells were exposed to 25 mM EtOH for two days or one week and the levels of γH2AX and 53bp1 were measured by Western blotting. (I) A representative example of the γH2AX Western blot is shown. + marks a positive control of cells exposed to Doxorubicin for two days. (II) A representative example of 53bp1 Western blot; (III) Quantitation of II. * pV < 0.05 ** pV < 0.01. (**D**) Telomere length (TRF) in different cell types exposed to AcH, 4-MP or recovered from EtOH treatment. Cells were exposed to AcH, 4-MP and the length of their telomeres was assessed by Southern blot. (I) A representative example of a Southern blot measuring TRF length. (II) Quantitation of TRF measurements. Percentages represent relative TL length to control untreated samples. * pV < 0.05 **pV < 0.01.

In light of the high increase in XPF binding to telomeres, we assessed its involvement in telomere shortening by EtOH by downregulation of its expression in the cells. Unexpectedly, the knockdown resulted in a massive death of transfected cells (Figure 4A), suggesting that the XPF gene product is essential for their survival. This essentiality is probably related to its function as a DNA repair factor but needs to be further explored.

### 3.7. The Role of PARP1 in EtOH-Dependent Telomere Shortening

As above, the marked change in the binding of PARP1 to telomeres in response to EtOH motivated us to study its involvement in EtOH-dependent telomere shortening. For that purpose, a chemical inhibition was performed with two PARP1 pharmacological inhibitors, 3-AB and IQD. Drugs concentrations that killed >10% of the cells were chosen for the analysis of telomere lengths in response to PARP1 inhibitors. As shown in Figure 4B, PARP1 inhibition abolished the EtOH-dependent telomere shortening observed throughout the study. Along these lines, exposure to 3-AB or IQD in addition to EtOH ethanol resulted in 13% and 5% increase in average TRF length, respectively.

### 3.8. The Presence of DNA Double-Strand Breaks Was Not Apparent

TRF2 binds and inhibits ATM, a key protein in the cellular response to double strand breaks (DSB). Therefore, the presence of γH2A.X was assessed in all three cell lines (Figure 4C). However, no evidence of this phosphorylated histone was observed in response to 25 mM EtOH treatment for two days or one week. In addition, we assessed the cellular levels and the level of binding of 53bp1, another DNA repair protein, to telomeres in response to EtOH treatment. 53bp1 cellular levels have decreased by ~20% relative to the control (untreated) cells and by ~30% after a week of exposure. Analyses of the ChIP results demonstrated a significant decrease of 90% in p53BP1 binding to TLs after a week of exposure compared to the control cells, a change that has been seen already after two days of exposure (Figure 4D). However, these observations do not exclude the formation of DSB in response to ethanol exposure.

### 3.9. Acetaldehyde May Have a Key Role in TL Shortening in Response to EtOH Exposure

In order to decipher the mechanism underlines TL shortening in response to EtOH treatment we focused on its downstream metabolism in the body and cells as an alternative approach. Accordingly, the cells were exposed to three more treatments: 25 µM acetaldehyde: (AcH), the first EtOH metabolite in the human body [15], 2 mM 4-methylpyrazolean (4-MP), an ADH inhibitor and a recovery treatment in which the cells were given a standard EtOH treatment of 25 mM for one week followed by a withdrawal of EtOH from the growth medium and providing the cells with fresh medium for an additional week.

Mean TRF was measured as before in all cells exposed to the abovementioned treatments. The results (Figure 4D) demonstrated that AcH promoted TL shortening in all cell lines, with an even bigger effect than that of EtOH. In the two fibroblast cell lines, 4-MP treatment decreased the extent of TL shortening in response to EtOH compared to EtOH treatment alone. However, this effect was not observed in the HepG2 cells. Recovery treatment promoted relatively longer TLs than EtOH-treated ones in immortal cell lines HepG2 and in hFF-IM. This implies that the recovery period has allowed the shortened TLs to be elongated by telomerase. The extent of TL shortening in both fibroblast cell lines was much higher than that of HepG2, probably due to the latter’s tumorous nature with extremely short TLs, characteristic of cancer cells.

### 3.10. Epigenetic Changes in Response to EtOH

**Figure 5:**
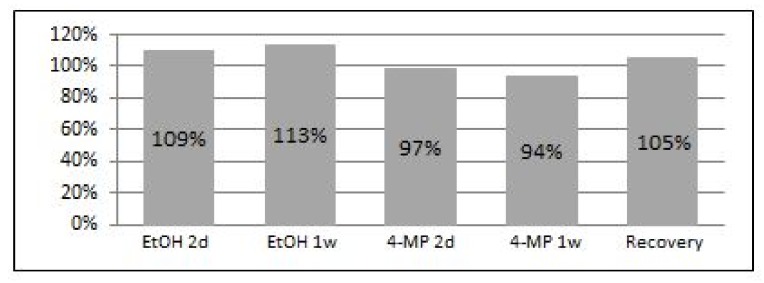
Methylation analysis of sub-telomeric regions of chromosome 10 in hFF-IM cells. Cells were treated with both EtOH and 4-MP for two days and one week, and additionally with the recovery treatment. Analysis of methylation status was performed by pyrosequencing. Percentages are relative to untreated control.

To clarify a possible involvement of changes in the epigenetic status of subtelomeric regions in our setting, we analyzed the levels of methylation in these regions at chromosome 10 using pyrosequencing (Figure 5). hFF-IM were subjected to two days and one week of both EtOH and 4-MP treatments, as well as a recovery treatment.

The status of the sub-telomeric methylation correlated with the time of exposure of the cells to EtOH: the longer the time of cells exposure to EtOH was, the higher the extent of their sub-telomeric regions’ methylation become. Methylation levels also correlated with the TL length: cells provided with the recovery treatment and therefore elongated their telomeres showed lower methylation levels at their subtelomeres compared to the levels in cells exposed to week or even two days of EtOH. However these recovered cells exhibited higher levels of methylation than that of the control. Cells treated with the EtOH inhibitor 4-MP did not exhibit hypermethylation but rather a degree of hypomethylation.

### 3.11. Changes of Genome-Wide Expression in Response to EtOH Treatment

**Figure 6:**
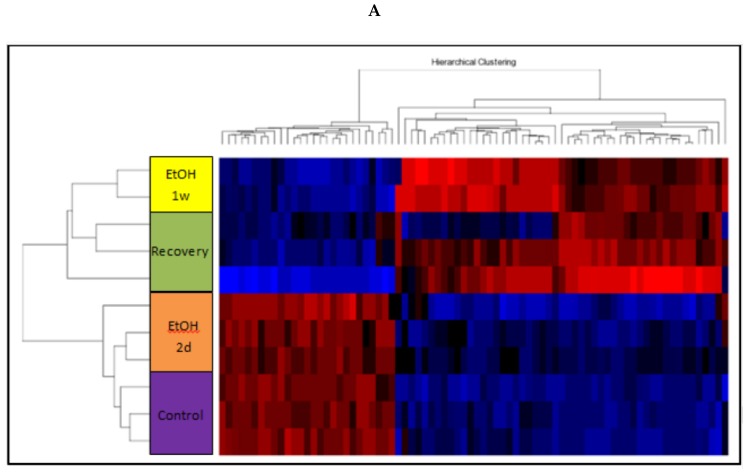

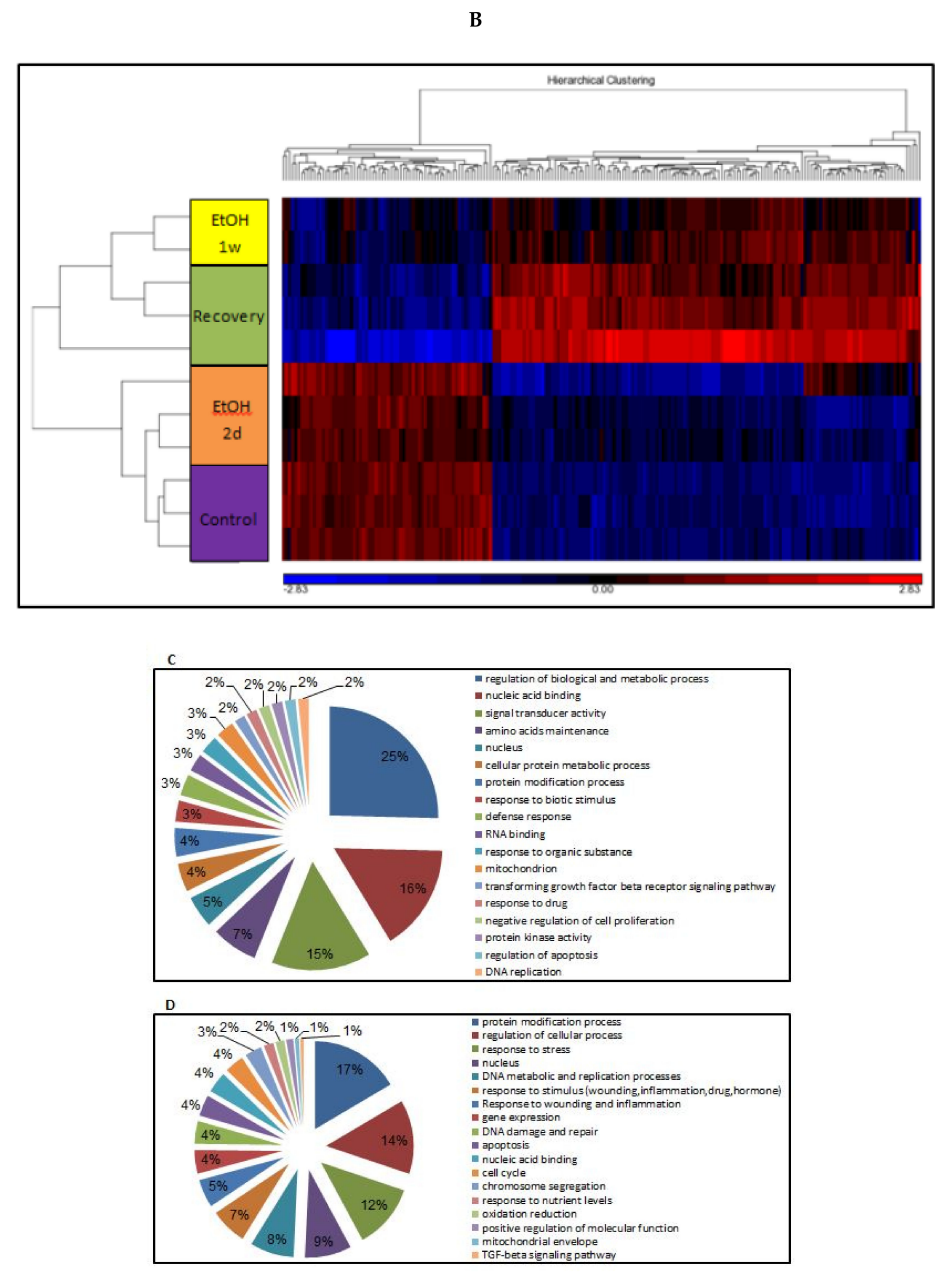

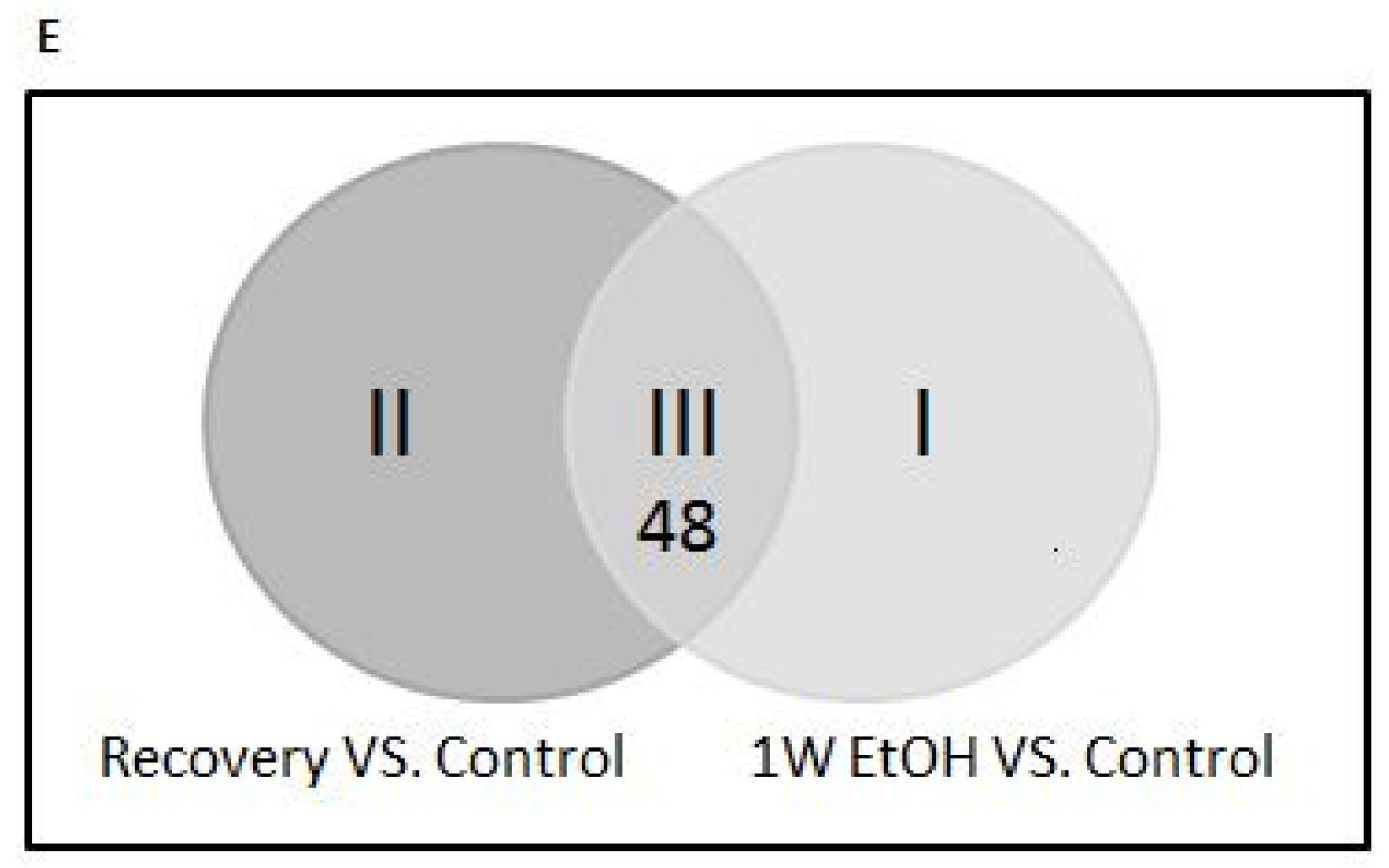
Functional analysis of gene expression in response to EtOH treatment and the recovery from EtOH insult. Cells were exposed to 25 mM EtOH for one week and the expression of their gene expressions was analyzed by microarrays hybridization. Function analysis was done by the DAVID algorithm. (**A**) 25 mM EtOH treatment of one week (78 genes). (**B**) Recovery treatment (207 genes). (**C**) Hierarchal clustering of gene groups in cells treated with 25 mM EtOH for one week (78 genes). (**D**) Hierarchal clustering of gene groups in cells treated with 25 mM EtOH for one week and then recovered for additional week (207 genes). (**E**) Overlap of gene expression between two difference treatments: exposure to 25 mM EtOH and the recovery post EtOH treatment. Gene expression analysis results of the two groups that changed their expression in response to EtOH and recovery treatment were subjected to Venn diagram analysis. The cutoffs of pV < 0.05 and fold change differences of at least 1.5, with a false discovery rate (FDR) below 0.05, were used. (I) Genes whose expression levels were significantly changed only after EtOH treatment. (II) Genes that only responded to recovery treatment. (III) Genes exhibited a significant change in expression levels in both treatments.

To further study the molecular changes occurred in response to EtOH exposure, cells were treated with 25 mM EtOH for two days, one week or recovered from one week of EtOH treatment as above. Analyses of the gene expression profiling of the various samples revealed three distinct groups: the untreated control, one week of 25 mM EtOH, and recovery treatment. (Exposure of 25 mM EtOH for two days resembled the control) (Figure 6A,B)

The fact that both hierarchical clusterings (Figure 6D,E) have shown a similar pattern strengthens the validity of the proposed distance among the three groups from the untreated control group. Cells exposed to EtOH for one week differed the most from those of the control group, recovery treatment cells showed a medium distance to the EtOH and the two-day treatment is the closest of all to the controls.

Between the EtOH-treated cells and the recovered ones, two sets of differentially expressed genes were found: 78 and 207 genes of one-week EtOH and recovery treatments, respectively.

Functional analysis by DAVID algorithm of the two sets of genes depicted a different distribution pattern among the expressed genes: while the first three functional groups of the EtOH treatment are attributed to regulation of biological and metabolic process (25%), nucleic acid binding (16%) and signal transducer activity (15%), in the recovery treatment most genes that changed their expression are related to protein modification process (17%), regulation of cellular process (14%), followed by genes related to stress response (12%) and nucleus (9%; Figure 6C).

## 4. Discussion

In light of a plethora of studies describing the effects of various environmental stresses on the dynamics of telomeres [33,34,38,39], this study aimed at exploring the effect of EtOH, a common stress agent, on the TL dynamics of human cells in vitro in a controlled experimental system.

The main finding of our study is that moderate EtOH concentrations, similar to social drinking concentrations, shortened telomeres in numerous different cell types. Interestingly, a recent study has shown that alcoholic patients had shortened telomere lengths, placing them at greater risk of age-related illnesses, such as cardiovascular disease, diabetes, cancer and dementia [40]. Treating the cells with EtOH’s first metabolite, AcH, caused similar TL shortening in all cell lines; conversely, the inhibition of EtOH oxidation to AcH by 4-MP almost diminished the EtOH-dependent telomere shortening in hFF cells. Although the 4-MP inhibition was anticipated to be effective mainly in HepG2 cells, its effect in fibroblasts may be attributed to its Km, which is similar than that of cells in the human liver [41] compared to the relatively low activity of ADH in HepG2 cells [42].

The observations of TL shortening after AcH treatment on the one hand, and the reduction in TL shortening after the inhibition of AcH metabolism on the other hand, imply that AcH, rather than EtOH itself, is responsible for that TL shortening effect. Following this assumption, the minor shortening of TLs in fibroblasts treated with 4-MP can be explained by elevated levels of AcH in these cells as well, due to the activity of the alternative pathways of EtOH oxidation carried out by CYP2E1 and catalase.

AcH is known to be an oxidative stress generator. As its main target is the mitochondria, AcH imposes a multifactorial challenge on the redox system by enhancing the activity of NAD(P)H oxidase, which generates superoxide as well as reduces the ability of antioxidant enzymes such as glutathione to bind their ROS substrates to form DNA adducts [43]. Additionally, AcH can bind directly to the DNA to form DNA adducts [44], which induces the formation of lesions ranging from point mutations to DNA inter-strand cross-links and sister chromatid exchanges, thus impairing DNA replication resulting in carcinogenesis [44].

Telomere length is canonically regulated by telomerase, which demonstrated no significant change in its activity in the setting of our study. However, TRF2 has increased its binding to telomeric regions in response to EtOH exposure. TRF2 is known to be involved in telomeric DNA protection against chromosomal end-to-end fusion by the induction of t-loops formation and their maintenance [45]. T-loop structure is known to inhibit DDR- and ATM-related processes but also to prevent telomerase from binding to the 3′ overhang of telomeres, thus serving as a negative regulator of TL’s length [46]. This is in line with the TA results, since the TRAP assay cannot provide data in *cis*, but only in *trans* as the active enzyme is purified from cells and its activity is assessed regardless of telomere structure (open or locked in t-loops conformation).

The observed increase in TL-bound TRF2 might be attributed to the presence of AcH (directly administered or metabolized by cells) and its harmful activities in the cell. The oxidative stress induced by EtOH or AcH may explain the observed EtOH-dependent TL shortening [47,48], where the actual mechanism underlying this shortening is generally attributed to DNA instability such as double- or single-strand breaks and the recruitment of DDR proteins. Similar, yet supported by little data in the literature, is the effect of DNA adducts on TL length.

All TRF2 binding proteins, involved in DNA repair processes increased their binding to telomeres in response to EtOH treatment, implying their putative involvement in telomere shortening. Of them, two exhibited the highest level of binding: PARP1 and XPF. The silencing of the expression of XPF was lethal to the cells, emphasizing its essentiality to their survival probably with regards to DNA repair-related processes. This observation was not shown in other studies; research published recently studying mitotic progression and multinucleation has shown that XPF knocked down in hepatocyte cells did not disrupt cells’ survival [49].

The inhibition of PARP1 activity attenuated the shortening of TL induced by ethanol, suggesting that PARP1 is at least partially involved in ethanol-dependent telomere shortening. However, a study describing accelerated aging during chronic oxidative stress showed that PARP-1 can reduce telomere shortening by enhancing DNA repair, contributing to telomere protection [50]. Another study showed genomic instability induced by the inhibition of PARP1 in telomerase-deficient mouse embryonic fibroblasts [51], connecting PARP1 to genome instability against the background of telomere dysfunction due to telomerase silencing. PARP1 involvement in our setting may be direct or indirect, providing a possible explanation for the above controversial results, an issue that may be clarified in future studies

We observed a change in the methylation status of the subtelomeric region of chromosome 10. Recent studies showed a correlation between the methylation status of sub-telomeric region and TL length [52]. Changes in the methylation status of these sub-telomeric regions may lead to the shortening of telomeres. For example, in Alzheimer’s disease cells in which the subtelomeres were hypermethylated shortened their telomeres [52].

All in all, it seems that several pathways converge to cause telomeres to shorten after EtOH exposure. These include the enhanced binding of TRF2 to telomeres, the subsequent binding of PARP1 and XPF1 and the changes in the methylation status of the sub-telomere regions.

Gene expression analysis showed that EtOH induced two types of expressed genes: one group exhibited changes after EtOH exposure, and the other set of genes was changed after the recovery of the cells from EtOH insult (Figure 6). Probably the mild telomere shortening that occurred in response to EtOH induced at least some of these irreversible changes. This highlights the long-term irreversible damages of EtOH consumption on cells that are not mended following recovery.

There are several pitfalls in our study. First, while both HepG2 and hFF-P cell lines have demonstrated a significant elevation of DNA-bound TRF2 levels, no change was observed in hFF-IM in response to EtOH exposure. The reason for this difference is still unknown and further study is needed to clarify this point.

In addition, the expression of γH2A.X and 53bp1, both known biomarkers for DSB [53], was not elevated in EtOH-treated cells. Similarly, the decrease of 53BP1 binding to TLs in response to ethanol does not exclude the existence of DSB along the DNA induced by ethanol, but possibly points to other DNA repair factors that are involved in the DNA damage and repair mechanism imposed by ethanol. These may include MDC1/TOPBP1 as both serve as markers for DSBs [54].

Although the AcH effect was not included in analyses of the genome-wide expression and the methylation level of sub-telomeric regions, these analyses can support the general assumption that EtOH induces stress upon exposure to cells. In addition, it would be interesting to use ALDH inhibitors in future research to provide another angle of observation regarding the AcH effect on TL dynamics.

To conclude, alcohol, as one of humanity’s most ancient and most used drugs, leads to both beneficial and harmful effects, which are commonly separated by the vague border of “moderate drinking” concentrations. This study provides evidence that, even at moderate concentrations, probably through the metabolism of ethanol to acetaldehyde, telomere erosion is being accelerated. As alcohol consumption is associated with ageing and cancer, understanding the downstream chain of events in this process may greatly contribute to future developments and treatment of aging-related diseases, including cancer.
